# Correction: Can immersive virtual reality magnify treatment outcomes of computerized script training on Cantonese speakers with chronic aphasia? Protocol of a randomized controlled trial

**DOI:** 10.1371/journal.pone.0357039

**Published:** 2026-08-26

**Authors:** Winsy Wing Sze Wong, Donald Shi Pui Li, Kenneth Nai Kuen Fong, Peter Hiu Fung Ng, Hoi Tsz Karen Kwok

The third author's name is spelled incorrectly. The correct name is: Kenneth Nai Kuen Fong.
