# Supplementary material for: Can immersive virtual reality magnify treatment outcomes of computerized script training on Cantonese speakers with chronic aphasia? Protocol of a randomized controlled trial
Source: PLoS One. 2026 Jun 2;21(6):e0350390. doi: 10.1371/journal.pone.0350390 (PMC13229358; doi:10.1371/journal.pone.0350390)
Supplement: S1 Protocol — (PDF) [file pone.0350390.s003.pdf]

Research Study Protocol (version 1.0; 24th July 2024)

Study Protocol Title:

Title: Can immersive virtual reality magnify treatment outcomes of script training on Cantonese speakers with chronic aphasia? A randomized controlled trial

Principal Investigator, Research Team, and Study Site:

Principal investigator: Dr. Wing Sze WONG
Co-Investigators: Dr. Donald Shi Pui Li, Dr. Kenneth Ngai Kuen Fong3, Dr. Peter Hiu Fung Ng4,
Study site: Speech Therapy Unit, The Hong Kong Polytechnic University

Research Synopsis

Study Title: Can immersive virtual reality magnify treatment outcomes of script training on Cantonese speakers with chronic aphasia? A randomized controlled trial

Study Population

Cantonese-speaking individuals with post-stroke aphasia

Study Design

A prospective, three-armed, open-label, assessor-blinded RCT will be conducted over a period of 20 weeks, based on a treatment protocol developed by the project team (Wong & Li, submitted). Protocol design and data reporting are aligned with the Consolidated Standards of Reporting Trials Statement (CONSORT) for nonpharmacologic trials (Boutron et al., 2017).
Trial Registration Data Set has been registered in ClinicalTrials.gov (**NCT06722092**).

Sample Size/inclusion/exclusion criteria

A total of 105 Cantonese PWA will be recruited from rehabilitation centers/support groups for stroke patients in Hong Kong, and the speech therapy clinics of the local institute. Sample size is based on a power analysis of 90% with α=.05, a medium effect size (which is considered conservative with respect to previous studies with comparable total time for treatment such as Cherney et al., [2014] and our pilot study (Wong et al., submitted), in which a large effect size was obtained), and an estimated attrition rate of 15%. Inclusion criteria include: 1) a stroke onset of more than six months, with an Aphasia Quotient (AQ) below 96.4, as evaluated by the Cantonese version of Western Aphasia Battery (CAB; Yiu, 1992), 2) premorbid fluent Cantonese speakers, 3) aged between 30 and 80 years, 4) no reported progressive neurogenic disorders such as dementia or Parkinson's disease, 5) no motor speech disorders of moderate to severe level, and 6) normal or corrected-to-normal vision and hearing functions. (R6) Recruitment through the collaborating rehabilitation centers and support groups for PWA will be done via posters and social media posts. Screening will be done by a trained research postgraduate student/research assistant with speech therapy background.

Study Duration

36 Months

Study Agent and Intervention Description

All of the treatment and generalization probes, language, and cognitive measures will be tapped twice before treatment three weeks apart as baseline. They will be subsequently administered mid-treatment, within a week post-treatment, as well as 8-week post-treatment as maintenance. The timeframe of the study is illustrated in Figure 1.

PWA who have given informed consent will be randomly allocated to one of the following conditions: 1) VR treatment condition, 2) Non-VR treatment condition, or 3) no-treatment condition, based on a computer-generated randomization sequence created by the co- investigator (Li) with an allocation ratio of 1:1:1. (R8) All participants in the no-treatment group will be entitled to VR/non-VR-based treatment upon the completion of all.

A total of 14 individual treatment sessions, given twice/three times per week, will be delivered to both treatment groups. Each session will include a 30-minute computerized script training, followed by a 30-minute practical section delivered with or without VR.

Computerized script training
A computerized script training program delivered in Cantonese, with procedures adopted from Cherney et al. (2008), will be used. The program has already been developed by the PI as supported by an internal grant provided by the institute (R2). Each treatment session begins with a session probe, which monitors the PWA's performance on scripts trained in previous sessions without support/cueing. If the performance on a certain script exceeds 90% in consecutive two sessions, a new script will be introduced. Treatment follows the sequence of: 1) The whole conversation script will be presented on a computer screen and read aloud by the virtual clinician, 2) The PWA will read each conversational turn assigned twice in unison with the virtual therapist. 3) The PWA will read aloud the sentence independently while the verbal response will be recorded by the program, 4) Treatment proceeds to another conversational turn. Each script will be repeated twice while two to three scripts will be trained in each session in random order. A clinical assistant will be present to monitor and provide technical support. Following computerized script training, the5 same sets of scripts will subsequently be practiced for 30 minutes, either in the VR or non-VR condition. The same set of procedures will be applied in the two treatment conditions; they only differ in terms of how treatment materials are presented.

VR treatment
The PWA will wear an HMD (e.g., Oculus Quest 2) for 30 minutes. Interactive 360-degree videos depicting various daily scenarios will be presented. (R2) The immersive experience provided by the 360-degree videos will include a visual exploration of the scenario/surroundings (e.g., walking around in a fashion shop/reading a food menu in a restaurant) and audio input via stereo speakers provided by the HMD (traffic on the street, background noise of a Chinese restaurant, etc.). Interactive components will be achieved mainly via communication with the virtual communication partner and the subsequent event taking place. For example, a waiter in the restaurant will initiate a conversation (see Figure 2b). The PWA will be given time to respond. If a correct response is produced, the virtual communication partner will 'interact' with the PWA by initiating the next conversational turn, and, if applicable, nonverbal reactions/rewards such as presenting the object requested to the PWA. For incomplete or erroneous productions, PWA will be required to repeat the correct response in unison with the recorded model. The clinical assistant, who accompanies the PWA in the entire training session, will decide and control the type of feedback. Rests within

the session will be given and any possible adverse effects will be monitored and reported.

Non-VR treatment
The flow of training is identical to the VR condition, except that only static photos depicting the scenarios and pre-recorded conversational turns will be presented to the PWA on a computer screen (Figure. 2c).

Research Questions
1. Can script training promote verbal functional communication of Cantonese-speaking PWA? With regard to the evidence established in English speakers with aphasia and our pilot study, our proposed RCT can provide empirical evidence for the intervention approach.
2. Can script training enhance nonverbal cognitive functions of Cantonese-speaking PWA?
3. Can VR magnify verbal and nonverbal treatment outcomes of script training of PWA?

Primary Objective

PWA's accuracy and time needed to produce the trained scripts (i.e., treatment probe) in read-aloud and no-cue conditions will be recorded via the computerized script training program. Similar to training, the virtual therapist will initiate the conversation, followed by PWA's response. The no-cue condition will be executed first, followed by the read-aloud condition. The performance of PWA receiving treatment on the session probe in terms of the number of sessions required to reach the performance criterion (i.e., 90% accuracy in consecutive two sessions) will also be considered.

Secondary Objectives

Secondary outcome on nonverbal cognition is comprised of a test battery measuring EF, short-term/working memory, and attention used in previous studies of PI that investigated the relation between nonverbal cognition and multi-level language processing (Wong & Law, 2022a) and their changes as a result of functional communication therapy (Wong & Law, 2022b). Attention is assessed via sub-tests 1 (map search) and 3 (elevator counting) of the Test of Everyday Attention (Robertson et al., 1994). The former aims to tap sustained and selective visual attention via identifying the target symbol in a map while the latter assesses auditory selective attention via counting the number of target tones in the presence of distractors with different tones. Verbal short-term and working memory will be examined via digit span forward and backward tasks from the Wechsler Adult Intelligent Scale – 4th edition (2008), respectively. EF will be evaluated via the Test of Nonverbal Intelligence-third edition (TONI-3; Brown et al., 1997), and the Wisconsin Card Sorting Test (WCST; Grant & Berg, 1993). TONI-3 is a norm- referenced test that uses abstract figures to evaluate abstract reasoning and problem-solving. WCST assesses cognitive flexibility and reasoning via matching cards with respect to color, quantity, and shape upon different rules and feedback given.
In accordance with the Research Outcome Measurement in Aphasia (ROMA; Wallace et al., 2018), the aphasia quotient of CAB, and CANELT (Wong et al., 2023), a standardized 6 measure adapted from the Amsterdam-Nijmegen Everyday Language Test (ANELT; Blomert et al., 1994), will be included as secondary outcomes in language and communication, respectively. CAB is a standardized aphasia test for measuring aphasia severity. CANELT assesses verbal communication effectiveness in conversation via 20 culturally appropriate scenarios. PWA's self-reported outcome on communication and quality of life will be examined via the Cantonese version of the 20-item

Communication Outcome after Stroke (Can-COAST) Scale (Kwok & Wong, 2023) translated from its original English version (COAST; Long et al., 2008). All assessment tasks and their sources of normative data (if applicable) are summarized in Table 1.

Adverse Event Reporting:

In case of a serious adverse event[1], please report to the Principal Investigator (Dr. Wong Wing Sze) immediately and the Principal Investigator will be required to report it to the PolyU IRB within 48 hours upon the receipt of your report.

[1]SAE is any adverse event that:

- Results in death
- Is life threatening, or places the participant at immediate risk of death from the event as it occurred
- Requires or prolongs hospitalization
- Causes persistent or significant disability or incapacity
- Results in congenital anomalies or birth defects
- Is another condition which investigators judge to represent significant hazards

(Reference: NIA Adverse Event and Serious Adverse Event Guideline)

Statistical Analysis Plan:

Assessment will be administered by a research postgraduate student with speech therapy background or trained speech therapy students who are blinded to the assignment of treatment condition. Scoring will be done by the research postgraduate student. Data analyses will be carried out by PI and Co-Is. Treatment and generalization probes will be scored by the Naming and Oral Reading for Language in Aphasia 6-point scale (NORLA-6) with some adaptations. NORLA-6 has been validated (Pitts et al., 2018) and adopted in a number of script training studies (e.g., Cherney et al., 2014; 2019). (R2) Adaptations in the current study concern the accuracy judgment of responses that semantically acceptable production will also be considered accurate during scoring. The time for script production will also be calculated. For secondary outcomes, PWA's performance on various cognitive tasks and CANELT will be transformed into z-scores. A principal component analysis will be carried out to group tests that share similar cognitive components. Linear mixed effect (LME) models will be used to investigate the research questions with timepoint and experimental conditions as fixed variables and different primary/secondary outcomes as predicted variables. Any significant results will be followed by post-hoc comparisons. Variables that may interfere with the outcomes such as age, aphasia severity, and education, will be included and controlled in the model.

Informed Consent Process:

Written informed consent will be sought from the participants with the presence of their caregivers.

Privacy and confidentiality:

1. No subject identifying information, other than recorded audio files of the subject's voice, will be recorded in any location other than the subject's signed consent form.
2. Code numbers will be assigned and used on all materials. Signed Informed Consent forms will be kept in a separate file from all coded materials.
3. Only the investigators and the team members of the current project will be permitted to access

the audio recordings, data, and consent forms. Furthermore, access to the data is password protected. Responsible members of The Hong Kong Polytechnic University may be given access for monitoring and/or audit of the research.

4. If the results of this study are published or presented in public, only transcripts with code numbers will be used.
5. At the end of three years after the completion of the study, all personal identifiers will be removed. However, anonymized data (including audio recordings) will be kept indefinitely for data sharing.

Risk/Benefit:

Risk to participants: The procedure has no known risks. However, it is possible that frustration or fatigue may be experienced by some subjects occasionally. Cyber-motion sickness may be experienced during VR-based training. You will have at least a 5-10-minute break every half-hour in each assessment/treatment session. You may take breaks more frequently if your wish. A session may be terminated at any point if you feel fatigued and unable to carry on.

Potential benefits: The research project will provide valuable information that will have important implications for assessment procedures, treatment design and monitoring of therapy progress in individuals with aphasia. Participants will have a better understanding on their performance on communication, language, and cognitive functions through assessment in the study period. Their communication/language/cognitive functions may benefit from training.

Study Timeline:

Stage 1, screening, enrollment: 1-26th months
Stage 2, data collection: 4-32nd months
Stage 3, data extraction and data analysis: 6-33rd months
Stage 4, data wrap up and report writing: 33-36th months

Data Safety Monitoring:

The PI will be responsible for data safety monitoring and reporting of any incompliance with the protocol/ delay in study schedule.

Conflict of Interest:

The applicants have no conflict of interest to declare.
