# Supplementary material for: Can immersive virtual reality magnify treatment outcomes of computerized script training on Cantonese speakers with chronic aphasia? Protocol of a randomized controlled trial
Source: PLoS One. 2026 Jun 2;21(6):e0350390. doi: 10.1371/journal.pone.0350390 (PMC13229358; doi:10.1371/journal.pone.0350390)
Supplement: Attachment — Submitted filename: Response_to_Reviewers(PONE-D-25-53607 & PONE-D-25-53607R1).docx [file pone.0350390.s005.docx]

Dear Editor,

**Response letter to the submission “Can immersive virtual reality magnify treatment outcomes of computerized script training on Cantonese speakers with chronic aphasia? Protocol of a randomized controlled trial” (PONE-D-25-53607 & PONE-D-25-53607R1)**

Thank you very much to the reviewers and editor for their comments and suggestions. We have considered them seriously and addressed them appropriately. Please find our point-by-point responses below.

Should you have any questions, please feel free to let us know.

Best,

Winsy Wing-Sze Wong (Corresponding author)

**PONE-D-25-53607R1**

Please upload a Response to Reviewers letter which should include a point by point response to each of the points made by the Editor and / or Reviewers. (This should be uploaded as a 'Response to Reviewers' file type.)

Response: The Response to Reviewers letter is uploaded to the system.

Please upload a copy of Figure 2

Response: The supporting information has been modified in the manuscript. In accordance with PLOS ONE editorial guidelines, the SPIRIT checklist (PDF) and SPIRIT Schedule (TIFF) have been uploaded as S1 Supporting Document and S1 Figure, respectively. Additionally, Protocol 1.0 has been uploaded as S1 Protocol in supporting information. Please note that Figure 2 is not included in the submission. Statement of fig2a is removed from line 303-304.

**PONE-D-25-53607**

**Major issues requiring attention**

1. **Randomization and allocation concealment**
   The protocol must provide a clear and explicit description of allocation concealment procedures, including who generates the randomization sequence, who enrolls participants, and who assigns interventions. Please also clarify whether stratified or minimization procedures will be used to balance key prognostic variables across the three arms, or justify why this is not planned.

Response: An explicit description of randomization and allocation concealment had been added under the Participant section (lines 187-204) and Randomization (lines 219-228). We did not plan to balance the prognostic variables (e.g., age, aphasia severity, and lesion size) in the condition assignment due to practicality issues. For instance, most of the stroke patients in Hong Kong did not have MRI scans post-stroke due to resource limitations. It is therefore impossible to consider all these variables during condition allocation. We fully understand their potential impacts on aphasia therapy outcomes; these variables (where available) will be included as covariates in the analysis, illustrated in the LME models (lines 385-443).

2. **Primary outcome definition and scoring procedures**
   The primary outcome requires more precise operationalization. Please clarify criteria for correct script production, handling of partial responses, self-corrections, and semantic substitutions.
   In addition, scoring procedures must be specified in greater detail, including the number of raters, blinding procedures, rater training, adjudication rules, and justification or validation of the adapted scoring approach (e.g., NORLA-6 adaptation).

Response: Scoring will be performed by the fifth author, trained speech-language pathology students, and trained personnel with backgrounds in speech-language pathology or linguistics (lines 345-349). Assessors will be blinded to the intervention condition. Patients will be reminded to keep their intervention condition confidential during assessment. Blinding procedures for assessment and treatment are detailed in lines 266-271.

Adaptation of NORLA-6 concerns the deletion of rules that are not applicable to Cantonese (including prosodic variation of rating 4 and verb tense use in rating 3; please refer to Table 3 for details). The remaining scoring criteria will be consistent with the NORLA-6 framework. Detailed scoring procedures and criteria are mentioned in lines 345-371.

3. **Reliability and double scoring**
   The current plan for double scoring a limited proportion of recordings requires strengthening. Please increase and pre-specify the proportion of recordings to be double scored, define acceptable reliability thresholds, and outline procedures if reliability falls below target.

Response: The proportion of recordings to be double-scored has been increased to 20%. The reliability threshold and contingency procedures for low-reliability are detailed in lines 468-471.

4. **Sample size calculation and assumptions**
   Please provide a fully transparent account of the sample size calculation, including justification for the assumed effect size, statistical test framework (e.g., LME vs. repeated-measures ANOVA), assumptions regarding correlations among repeated measures, and how power relates to specific planned comparisons. Attrition assumptions should also be clarified.

Response: Sample size estimation was conducted using G*Power for repeated measurements, within-between interaction ANOVA. Lines 187-204 detailed the sample size calculations and assumptions, including a clarified rationale for the expected attrition rate.

5. **Statistical analysis plan (pre-specification)**
   The statistical analysis plan requires further pre-specification, including the planned mixed-effects model structure, handling of baseline measures and covariates, treatment of missing data, sensitivity analyses, and approach to multiple comparisons. Planned subgroup or moderator analyses should be clearly identified as confirmatory or exploratory.

Response: Further details regarding the pre-specification of the statistical analysis, including the planned mixed-effects model structure, handling of baseline measures and covariates, treatment of missing data, sensitivity analyses, and approach to multiple comparisons, have been provided in lines 371-443. Subgroup analyses are exploratory (lines 426-427).

6. **Blinding and bias mitigation**
   Please clarify how assessor blinding will be maintained and monitored, and how potential expectancy effects related to unblinded treatment providers will be mitigated.

Response: As assessors will be blinded to the intervention condition, patients will be reminded to keep their intervention condition confidential during assessment. Blinding procedures for assessment and treatment are detailed in lines 266-271. Further details regarding bias mitigation have been addressed in lines 281-294.

7. **Treatment fidelity and therapist training**
   Additional details are required regarding therapist training, treatment manuals, fidelity monitoring procedures, and corrective actions for protocol deviations.

Response: Therapist training and treatment manuals will be monitored and developed by the authors (WW and HTK). Fidelity monitoring will be conducted by the author (WW) and an independent speech therapist, while the PI and the research team will oversee corrective actions for protocol deviations. These procedures have been addressed in lines 281-294 and lines 471-475.

8. **Safety monitoring and adverse events**
   The protocol should specify procedures for monitoring and reporting adverse events (including VR-related symptoms), criteria for discontinuation, and the structure of safety oversight (e.g., DSMB or equivalent justification).

Response: The project will follow safety guidelines issued by the Institutional Review Board of the Hong Kong Polytechnic University, based on the National Institute on Aging Adverse Event and Serious Adverse Event Guidelines lines 451-457. Specific procedures for adverse event monitoring have been further addressed in lines 457-463.

9. **Eligibility criteria and generalizability**
   Please clarify inclusion and exclusion criteria (including aphasia severity and motor speech disorders) and discuss how VR tolerance-based exclusions may affect generalizability.

Response: Criteria for defining the severity of aphasia and motor speech disorders are clarified (lines 205-207, 209-210). The study includes PWA with an aphasia quotient below 96.4 and no motor speech disorders of moderate to severe level according to the Therapy Outcome Measurement. The exclusion of PWA unable to tolerate 10 minutes of VR exposure may result in selection bias, which might impact the generalizability of the results. Detailed clarification of the inclusion and exclusion criteria can be found in lines 205-217. We agree that VR-based exclusions may affect generalizability, which has been included as a limitation of the study (lines 479-481).

**Additional comments**

In addition to the major points above, please address the minor and editorial comments from all reviewers, including clarifying timelines, outcome measurement details, data-sharing plans, adherence to reporting guidelines (e.g., SPIRIT), and language clarity.

Response: The above-mentioned comments have been addressed in response to the reviewer’s feedback (please refer to the specific responses below).
**Journal Requirements:**

1. When submitting your revision, we need you to address these additional requirements.

Please ensure that your manuscript meets PLOS ONE's style requirements, including those for file naming. The PLOS ONE style templates can be found at

https://journals.plos.org/plosone/s/file?id=wjVg/PLOSOne_formatting_sample_main_body.pdf and

https://journals.plos.org/plosone/s/file?id=ba62/PLOSOne_formatting_sample_title_authors_affiliations.pdf

Response: The manuscript has been edited according to PLOS ONE’s style requirements.

2. We note that the grant information you provided in the ‘Funding Information’ and ‘Financial Disclosure’ sections do not match.

When you resubmit, please ensure that you provide the correct grant numbers for the awards you received for your study in the ‘Funding Information’ section.

Response: The name of the funder has been corrected in the ‘Funding Information’ section.

3. Thank you for stating the following in the Acknowledgments Section of your manuscript:

We are grateful to the following organizations for their support in subject recruitment in the funding application: AKA Social Service, The Hong Kong Polytechnic University Speech Therapy Unit, The Hong Kong Stroke Association, The Neighbourhood Advice-action Council NT West Community Rehabilitation Day Centre.

We note that you have provided funding information that is not currently declared in your Funding Statement. However, funding information should not appear in the Acknowledgments section or other areas of your manuscript. We will only publish funding information present in the Funding Statement section of the online submission form.

Please remove any funding-related text from the manuscript and let us know how you would like to update your Funding Statement. Currently, your Funding Statement reads as follows:

Wong received the General Research Fund from Hong Kong Research Grants Council for the project (Project number: 15602024).

https://www.ugc.edu.hk/eng/rgc/funding_opport/grf/

The funders had no role in study design, data collection and analysis, decision to publish, or preparation of the manuscript

Please include your amended statements within your cover letter; we will change the online submission form on your behalf.

Response: Regarding the Acknowledgments section, we clarify that the mentioned organizations provided support solely for participant recruitment and were not involved in funding acquisition; amendments have been made to lines 511-514.

4. Please note that your Data Availability Statement is currently missing the repository name and/or the DOI/accession number of each dataset and a direct link to access each database. If your manuscript is accepted for publication, you will be asked to provide these details on a very short timeline. We therefore suggest that you provide this information now, though we will not hold up the peer review process if you are unable.

 Response: The links for the code and data have been added to the Data availability (lines 520-522) and Code availability sections (lines 525-526).

5. Your ethics statement should only appear in the Methods section of your manuscript. If your ethics statement is written in any section besides the Methods, please move it to the Methods section and delete it from any other section. Please ensure that your ethics statement is included in your manuscript, as the ethics statement entered into the online submission form will not be published alongside your manuscript.

 Response: The ethics statement has been relocated to the Methods section in lines 181-185.

6. If the reviewer comments include a recommendation to cite specific previously published works, please review and evaluate these publications to determine whether they are relevant and should be cited. There is no requirement to cite these works unless the editor has indicated otherwise. 

Response: Not applicable.
[Note: HTML markup is below. Please do not edit.]

Reviewers' comments:

Reviewer's Responses to Questions

**Reviewer #1:** For sample size calculation, what test was used, t-test? Was a repeated measure method considered?
Descriptive statistics can be added to the statistical analysis plan.
Was baseline considered as a covariate in the LME? Change from baseline can be used as the dependent variable.
P value adjustment needs to be clearly written.

Response: Sample size estimation was conducted using G*Power (version 3.1.9.7) for repeated measurements, within-between interaction ANOVA. The detailed calculation methodology is provided in lines 187-204. Descriptive statistics for outcome measures and characteristics of PWA have been added under the section of Data Analysis (lines 371-372). We agree that baseline performance should be included in the LME and each model is specified in lines 372-419. P-value adjustment has been clarified (lines 425-426).

**Reviewer #2:** The study is well-grounded, and the authors demonstrate a solid understanding of the subject matter and relevant literature. The work addresses an important need to strengthen the validity of studies conducted using virtual reality (VR).

However, several points warrant reconsideration:

Protocol Design and Covariates: The study design does not account for covariates such as age, severity, and time since stroke, and the power calculation was performed without considering these factors. Additionally, these covariates are not planned to be balanced between groups. This represents a significant limitation that could reduce the validity of the results.

Response: Due to practicality issues, we did not plan to balance the factors in condition allocation. We did not plan to balance the prognostic variables (e.g., age, aphasia severity, and lesion size) in the condition assignment due to practicality issues. For instance, most of the stroke patients in Hong Kong did not have MRI scans post-stroke due to resource limitations. It is therefore impossible to consider all these variables during condition allocation. We fully understand their potential impacts on aphasia therapy outcomes; these variables (where available) will be included as covariates in the analysis, illustrated in the LME models (lines:385-419).

Population Specificity: The study focuses on Cantonese-speaking participants. While this is appropriate from a study design perspective, it should be clearly acknowledged as a limitation in the manuscript.

Response: We thank the reviewer for raising this. Population specificity has been addressed in the limitation section (lines 477-478).

Subjective Assessments: Some subjective evaluations are conducted by a single author. It may be advisable for multiple evaluators to perform these assessments independently, followed by a joint review of scores, to improve reliability.

Response: We agree with the reviewer’s suggestions. Scoring and subjective evaluation will be conducted by the fifth author (HTK), trained speech language pathology students and trained personnel with speech language pathology or linguistics background (lines 345-349).

General Writing and Clarity: The manuscript has issues with overall fluency, and certain sections contain grammatical errors. A thorough revision of the text is recommended to improve readability and clarity.

Response: The manuscript has been proofread by a professional copywriter for editorial clarity.

Overall, the study has strong potential, but addressing the points above would enhance its rigor and the clarity of its presentation.

**Reviewer #3:** - Well-motivated, timely three-arm RCT (VR vs non-VR computerized script training vs no-treatment) targeting an important clinical question for Cantonese-speaking PWA. Trial is registered (NCT06722092), ethics approved (PolyU IRB), and data/code sharing on OSF is declared. The basic design and choice of script training are appropriate; however, several methodological, safety, and analysis details require clarification or strengthening before the protocol is acceptable.

Major issues (expanded)

1. Randomization and allocation concealment
- Provide explicit allocation concealment procedures: who generates and stores the sequence, who enrolls participants, and who performs assignments. Describe physical/technical safeguards (e.g., opaque sealed envelopes, central web-based randomization).

Response: Our co-author, DL, who is not involved in any participant contact activities, will prepare a central web-based randomization sequence. Enrollment will be performed by co-author HTK. Condition assignment will be managed by author WW and a trained research assistant who is not involved in enrollment, screening, or assessment. Explicit allocation concealment procedures are addressed in lines 219-228.

- Consider stratified randomization or minimization on key prognostic variables (baseline AQ/aphasia severity, time since stroke, age) to prevent imbalance across three arms.

Response: Due to practicality issues, prognostic variables are not planned to be balanced; instead, simple randomization will be used (lines 225-228). We agree that some key variables may affect the evaluation of treatment outcomes. These variables will be included as covariates in the data analysis (lines 385-419).

2. Primary outcome definition, scoring, and rater procedures
- Fully operationalize the primary outcome: precise criteria for “correct” script production (treatment of semantic substitutions, paraphasias, partial utterances, prompts), timing window, and handling of self-corrections.
- Provide details on scoring procedures: blinded independent raters, number of raters per recording, training, and adjudication rules.
- The NORLA-6 adaptation and counting “semantically acceptable” productions as correct require justification or validation evidence (pilot reliability/validity). If novel, include plan to validate scoring (inter-rater reliability targets, pilot ICCs).

Response: Scoring will be performed by the fifth author, trained speech-language pathology students, and trained personnel with backgrounds in speech-language pathology or linguistics (lines 345-347). Assessors will be blinded to the intervention condition. Patients will be reminded to keep their intervention condition confidential during assessment. Blinding procedures for assessment and treatment are detailed in lines 266-271. Scoring rules that are not applicable to Cantonese have been deleted from the marking criteria. The remaining scoring criteria will be aligned with the NORLA-6 framework. Detailed scoring procedures and criteria are mentioned in lines 350-371.

3. Reliability and proportion of double scoring
- Ten percent double-scoring is low. Increase to at least 20–25% of recordings, sampled across arms, timepoints, and script types. Pre-specify acceptable ICC thresholds and procedures if reliability is below threshold (retraining, rescoring).

Response: 20% of data will be examined by the research postgraduate student and an independently trained speech therapist. Threshold and procedures are listed in lines 468-471.

4. Sample size calculation and assumptions
- Report full sample-size inputs: effect size origin and justification for d=0.74, assumed correlation among repeated measures, number of repeated measures, alpha adjustment for multiple pairwise comparisons (if applicable), and statistical test basis (LME vs repeated measures ANOVA). Clarify whether power is for VR vs non-VR, VR vs control, or all comparisons.
- Explain how the 20% attrition was applied and whether recruitment targets account for missing-at-random vs differential dropout across arms.

Response: Sample size estimation was conducted using G*Power for repeated measurements, within-between interaction ANOVA. Sample-size calculation and the rationale of attrition rate estimation have been clarified in lines 187-204.

5. Statistical analysis plan (pre-specify)
- Provide detailed LME specification: random intercepts and/or slopes, whether time is categorical or continuous, covariance structure, fixed effects (arm, time, arm*time), planned covariates (age, baseline AQ, education) and rationale.

Response: Detailed LME specification has been provided in lines 377-443.

- State handling of missing data (ML via LME, multiple imputation), sensitivity analyses, and assumptions to be tested.

Response: Handling of missing data (lines 425-426) and sensitivity analysis (lines 434-443) are addressed.

- Predefine primary contrasts and multiple comparison correction approach (hierarchical testing vs Bonferroni). Specify planned subgroup/moderator analyses (aphasia type, severity) and whether they are exploratory.

Response: Details on multiple comparisons and their nature (exploratory) are added (lines 425-426).

6. Blinding and bias mitigation
- Describe concrete steps to maintain assessor blinding (separate staff, standardized scripts to avoid revealing arm, blinding checks), and plan to assess and report blinding success.
- Clarify that therapy deliverers cannot be blinded and how expectancy effects will be mitigated (standardized instructions, scripted feedback).
Response: Assessors will be blinded to the intervention condition, while patients will be reminded to keep their intervention condition confidential during assessment. Clarification regarding unblinded therapy deliverers has been added (lines 281-282) and procedures to reduce bias mitigation has been added (lines 283-294). Concrete steps for assessor blinding are detailed in lines 266-271.

7. Treatment fidelity and therapist training
- Provide details on manuals, training sessions for therapists/assistants, fidelity checklists, frequency of fidelity audits, and predefined corrective actions if deviations occur. Specify who monitors fidelity (independent reviewer) and their criteria.
Response: Treatment fidelity will be monitored by the first author and an independent speech language pathologist (lines 471-475). Training sessions, standardized training with a developed protocol and therapy manual will be provided by the authors (WW and HTK). Detailed procedures are mentioned in lines 283-294.

8. Safety monitoring and adverse events
- Specify adverse-event monitoring procedures: which events are tracked (cybersickness, headache, dizziness, emotional distress), how often assessed, reporting timelines, criteria for temporary/permanent discontinuation, and who adjudicates events.
- State whether a DSMB or safety oversight committee will be convened; if not, justify and outline alternative safety oversight.

Response: A DSMB/safety oversight committee will not be convened; instead, the project will follow safety guidelines from the Institutional Review Board of the Hong Kong Polytechnic University, based on the National Institute of Aging Adverse Event and Serious Adverse Event Guidelines (lines 451-457). Specific procedures for adverse event monitoring have been further addressed in lines 457-463.

9. Inclusion/exclusion and generalizability
- Excluding anyone intolerant to a 10-minute VR exposure introduces selection bias toward those tolerating VR; discuss implications for generalizability and how findings will be interpreted relative to broader PWA populations.
- Clarify lower AQ limit (severe aphasia allowed?) and operational criteria for excluding “moderate to severe” motor speech disorders.

Response: The study includes PWA with aphasia quotient below 96.4 and no motor speech disorders of moderate to severe level according to the Therapy Outcome Measurement (lines 205-211). Previous studies did not show a clear indication regarding the lower limit of AQ in script training. As the first protocol on script training conducted in the Cantonese-speaking population, we opt not to set any lower AQ limit as an exclusion criterion. Participants are included provided they pass all the inclusion criteria during screening phase. We agree that, however, the exclusion of PWA who are unable to tolerate 10 minutes of VR exposure may result in selection bias, which might impact the generalizability of the results. This issue has been addressed as a limitation of the study (lines 480-481).

Minor issues and reporting suggestions

1. Sample and timeline consistency
- Reconcile inconsistent recruitment/start dates (May vs Aug 15, 2025) and provide final clear timeline. Provide updated, high-resolution SPIRIT figure
Response: Timeline has been updated in lines 483-485. High-resolution SPIRIT figure is provided.

2. Outcome measurement details
- For CANELT z-scores and other transformed measures, specify normative data sources and transformation procedures.

Response: CANELT (Wong, 2024) has been previously validated and published. The raw scores will be transformed to z-scores based on the normative dataset collected from 100 Cantonese speakers aged between 30-79 years old while raw scores will be used in the remaining measurement (lines 369-372).

- Clarify use of averaged baseline (two pre-treatment assessments) vs single baseline value in analyses.
Response: We propose implementing the pre-treatment assessment twice for establishing a stable baseline. To examine this, a repeated-measure ANOVA will be used to examine if the two baseline measurements are comparable. Outcomes with comparable baseline performance will be averaged across the two timepoints for subsequent analyses; conversely, the timepoint with better performance will be treated as baseline for subsequent analyses. Clarifications have been made under the section of Data analysis (lines 372-377).

3. Data sharing and reproducibility
- Good to see OSF links. Specify exactly which materials will be shared (de-identified audio, transcripts, scoring sheets, analysis code), timing of release, and de-identification steps for audio files.
Response: The links for the code and data have been added to the Data availability (lines 520-522) and Code availability sections (lines 525–526).

4. Reporting and adherence to guidelines
- Ensure completed SPIRIT checklist and adherence to CONSORT extension for non-pharmacologic trials and ROMA recommendations are demonstrated in the final protocol.
Response: Response: We have ensured that the study protocol adheres to the reporting standards as follows:

- SPIRIT Checklist: A completed SPIRIT checklist has been included as a supporting information file (S1 Checklist).
- CONSORT Extension: The study design aligns with the CONSORT extension for non-pharmacologic trials. The full CONSORT checklist will be provided during the results reporting phase. (lines 175-178)
- ROMA Recommendations: We have integrated the Rehabilitation Measures in Aphasia (ROMA) recommendations into our outcome measure selection (lines 246-257). Specifically:
  - Language Ability: Measured via the Cantonese version of the Western Aphasia Battery (CAB).
  - Communication: As the Scenario Test is not validated for the Hong Kong context, the Cantonese version of the Amsterdam Nijmegen Everyday Language Test (CANELT), a standardized outcome measure for communication among Cantonese speakers, is utilized as a secondary outcome for functional communication.
  - Quality of Life: For self-reported outcomes, the Cantonese Communication Outcome after Stroke (Can-COAST) will be administered to assess the participants' perceived communication ability and quality of life.

5. Figure/table quality and minor edits
- Improve figure resolution and correct small typographical repetitions in submission metadata.

Response: A high-resolution figure is provided in the submission system.
